# Physical Activity and Sedentary Behavior Patterns Prior to and During the COVID-19 Pandemic

**DOI:** 10.1093/geroni/igab046.1307

**Published:** 2021-12-17

**Authors:** Nancy Gell, Dori Rosenberg, John Bellettiere

## Abstract

Understanding patterns in the types of activities older adults engage in during physical activity and sedentary time could help shape intervention designs. Few studies have adequately described the physical activity and sedentary pursuits older adults undertake, including during the COVID-19 pandemic. To answer these questions, this symposium uses data from three recent studies: Adult Changes in Thought (ACT),an epidemiologic study with self-reported and device-based measures of physical activity and sedentary time including time spent in various domains of activity; Objective Physical Activity and Cardiovascular Disease Health in Older Women (OPACH), an epidemiologic study with device and self-report measures of sedentary behavior; and an ongoing clinical trial, the Healthy Aging Resources to Thrive (HART) study with device and self-reported data on sitting time and patterns as well as physical activity. The first session in this symposium will present a description of the rates of meeting the aerobic, strength, and balance recommendations among older adults in the ACT study. Next, we will have a presentation describing sedentary activities in older adults by age, sex and device-based sitting patterns in the ACT study. In the third presentation we will use OPACH data to examine patterns and context of sedentary in relation to aging-related outcomes. Finally, we will describe changes in physical activity and sedentary time in the HART trial in the cohort enrolled prior to the COVID-19 pandemic vs. those enrolled during the pandemic. Our Discussant will provide new insights on the roles of sedentary behavior and physical activity in aging and health.

